# Data on cross-border exposures of 61 largest European banks

**DOI:** 10.1016/j.dib.2020.105613

**Published:** 2020-04-23

**Authors:** Patty Duijm, Dirk Schoenmaker

**Affiliations:** a Rotterdam School of Management, Erasmus University Rotterdam and De Nederlandsche Bank; b Rotterdam School of Management, Erasmus University Rotterdam and CEPR

**Keywords:** Cross-border banking, Geographical diversification, Internationalization, Financial globalization

## Abstract

This article introduces a unique and hand-collected dataset on cross-border exposures of 61 European banks. Getting a complete overview of the cross-border positions of European banks is challenging, as there are no regular reporting standards for banks’ foreign exposures split by country. Most studies therefore rely on data on banks’ foreign subsidiaries. This however leads to a significant underestimation of banks’ cross-border positions. We collect data from annual reports and other public sources for the period 2010-2017 in order to construct a dataset covering the complete cross-border exposures by banks. The dataset is valuable to academic researchers in finance and economics as well as central banks interested in financial globalization. The data are collected at the individual bank-level, and this provides opportunities for researchers aiming to analyse the impact of banks’ strategic decisions [1]. Lastly, since the cross-border exposures are split by host country the data can be used in gravity models, since it provides a measure of connectedness between banks and/or countries.

Specifications Table

**Table:** 

Subject	Finance
Specific subject area	This dataset captures data on cross-border banking. It contains detailed information on the foreign activities by the largest European banks.
Type of data	Table (Excel format)
How data were acquired	Hand collected from public online sources, of which bank annual reports
Data format	Raw
Parameters for data collection	We collected data on cross-border exposures of European banks, and focussed on banks with total assets of EUR 100 billion in either 2017 or 2010.
Description of data collection	Hand collected from public online sources, of which bank annual reports.
Data source location	Europe (European banks)
Data accessibility	Repository name: Mendeley Data DOI:10.17632/k63trwdfmk.1 URL: https://data.mendeley.com/datasets/k63trwdfmk/1
Related research article	Duijm, P. and Schoenmaker, D. (2020). European Banks Straddling Borders: Risky or Rewarding? *Forthcoming in Finance Research Letters*

## Value of the data

• This dataset provides a complete picture of European banks’ cross-border exposures, whereas other public datasets on banks’ cross-border activities are often limited to data on banks’ cross-border exposures via its foreign subsidiaries, leading to a significant underestimation of banks’ cross-border positions [2].
• Academic researchers in finance and economics as well as central banks interested in financial globalization or cross-border activities of individual banks will benefit from this data.
• The cross-border exposures at bank-level provided by this dataset can be used to analyze the effects of bank internationalization.
• The cross-border exposures are moreover split by host country and can as such be used in, for example, gravity models since it provides a measure of connectedness between banks and/or countries.
• Complete data on cross-border banking in the European banking sector is especially relevant in light of the ongoing financial integration within the European Union.

## 1 Data Description

The dataset [3] is represented in an Excel file and contains bank-specific data on banks’ cross-border positions, split by a bank's host countries. Each bank is identified by a bank number, bank name, home country and a SNL code. The SNL code should be used as a key when merging the dataset with bank-specific financial statement data from the SNL Financial Database. The dataset is limited to European banks with total assets of EUR 100 billion in either 2017 or 2010. Only the Belgian bank Dexia and the German bank WestLB are left out, as with the restructuring of Dexia in 2010, a large part of the portfolio is now with Belfius bank, while Dexia operates as a “bad bank”. WestLB was split into three parts (of which one was a bad bank) in 2012 and significantly decreased its assets since then. For each bank and each year, we report the cross-border exposures split by host country whereas host countries are labelled by the ISO2-code. The exposures towards a certain country are expressed as a share of banks’ total exposures.

## 2 Experimental Design, Materials, and Methods

Data on cross-border positions are primarily obtained from annual reports, and, when needed, supplemented with data stemming from the public EBA stress tests conducted in 2011 and 2013, and country-by-country reporting, which is mandatory under the Capital Requirements Directive of 2013 (CRD IV). We have collected data for the period 2010-2017, as these latter two data sources are only available more recently. Table 1 below contains an overview of the source(s) used by bank.

**Table 1:** Data source and non-allocated data by bank.

Name	Exposure	Source	Not allocated	Name	Exposure	Source	Not allocated
HSBC Holdings	L	AR	0.0%	Swedbank	A	AR	4.5%
BNP Paribas	L, NI	AR, ST	3.1%	Landesbank Baden-Württemberg	A	AR, ST	0.0%
Crédit Agricole Group	A, NI	AR, CbC	0.0%	La Banque Postale	L	AR	0.8%
Deutsche Bank	L, NI	AR, CbC	0.0%	Bayerische Landesbank	A	AR, ST	8.3%
Barclays	L, NI	AR, CbC	0.0%	Banco de Sabadell	L, A	AR	0.8%
Banco Santander	L, A	AR	11.0%	Bankia	A	AR	0.3%
Société Générale	A, NI	AR, CbC	0.0%	Erste Group Bank	A	AR	2.7 %
Groupe BPCE	A, NI	AR, CbC	0.0%	Raiffeisen Gruppe Switzerland	L	AR	0.0%
Royal Bank of Scotland Group	A	AR	0.0%	Nykredit Holding	L,A	AR, ST	0.0%
Lloyds Banking Group	A	AR, CbC	0.6%	Norddeutsche Landesbank Girozentrale	A	AR, ST	0.0%
UBS Group	L, NI	AR, CbC	0.0%	Belfius Banque	A	AR	2.2%
UniCredit	L, NI	AR, CbC	4.4%	Landesbank Hessen-Thüringen Girozentrale	NI	AR, CbC, ST	0.0%
ING Bank NV	A	AR	0.0%	Banca Monte dei Paschi di Siena	A, NI	AR, CbC	0.1%
Credit Suisse Group	L, A	AR	0.0%	Banco Popular Español	A	AR	0.0%
Banco Bilbao Vizcaya Argentaria(BBVA)	A, NI	AR, CbC	0.0%	NV Bank Nederlandse Gemeenten	A	AR	0.0%
Crédit Mutuel Group	A, NI	AR, CbC	0.0%	Zürcher Kantonalbank	A	AR	0.0%
Intesa Sanpaolo	L, NI	AR, CbC	0.0%	NRW Bank	A	AR	8.1%
Coöperatieve Rabobank	L, NI	AR, CbC	0.0%	Raiffeisen Zentralbank Österreich	L	AR	1.7%
Nordea Bank	A, NI	AR, CbC	0.0%	Bank of Ireland	A	AR	1.9%
Standard Chartered	A, NI	AR, CbC	0.0%	OP Financial Group	A	AR	1.0%
Commerzbank	A, NI	AR, CbC	3.6%	Volkswagen Financial Services	A	AR	0.0%
KfW Gruppe	L	AR	0.0%	Banco Popolare Società Cooperativa	A	AR	0.1%
Danske Bank	NI	AR	3.1%	Unione di Banche Italiane	A	AR	0.1%
Deutsche Zentral-Genossenschaftsbank	NI	AR, CbC	0.0%	SNS Reaal	L	AR	0.0%
ABN AMRO Group	A, NI	AR	0.0%	National Bank of Greece	L, NI	AR, CbC	0.0%
CaixaBank	A	AR	1.0%	DekaBank Deutsche Girozentrale	A	AR	0.5%
Svenska Handelsbanken	A	AR	3.3%	Allied Irish Banks	L	AR	1.6%
Skandinaviska Enskilda Banken	A	AR	9.0%	Caixa Geral de Depósitos	A	AR	0.0%
DNB ASA	L	AR	0.0%	HSH Nordbank	A	AR, ST	0.0%
Nationwide Building Society	L, NI	AR, CbC	0.0%	Landesbank Berlin	A	AR	3.3%
KBC Group	A	AR	0.0%				

This table shows per individual bank the type of cross-border exposure and the source the data is based on as well as the percentage of total exposures that could not be allocated to a certain country or region. The following abbreviations are used:

Exposure: A = Assets, L = Loans, NI = Net Income

Due to the absence of a standard reporting format some assumptions and simplifications had to be made. First, while some banks report their foreign exposures in loans or assets, some banks use the net income as the reporting unit. As we are especially interested in banks’ credit exposures to other countries, we had an order of preference for exposures reported in i) loans; ii) assets; and iii) net income. Table 1 shows the type (loans, assets or net income) of cross-border exposure used by bank. The reason for our preference for loans and assets, is that these capture the real risk (loss of principal) the bank is exposed to. Asset and loan exposures can be considered quite similar, i.e. most of the assets reported to a specific country will be invested in the economy via (loans granted by) banks, government etc. Asset and loan exposures reflect the structural nature of cross-border banking. Income can be regarded as more different and volatile. However, only for three banks we rely solely on net income. For some of the other banks, we use the reported net income (from the country-by-country report) in situations where a bank reports a less granular geographical split of its assets or loans (e.g. “assets in Africa”). In that case, we use the net income information from the country-by-country report to subdivide the total asset exposure to Africa (on the basis of net income) to different African countries listed in the country-by-country report. As such, we aim to minimize measurement errors that results from using different measurements across banks.

Second, we aimed for cross-border exposures at the country level as for our analysis we link home and host country characteristics. However, sometimes only information on banks’ exposures to a group of countries (e.g. Western Europe) or continents (e.g. Asia) was available. In those cases where we could not further subdivide these grouped exposures, we simply collected the exposures to groups of countries or continents. For the analysis, we defined country characteristics – such as GDP per capita or unemployment - at a group or continent level by taking the (GDP weighted) average of all countries belonging to that group or continent.

Third, the data collection resulted in an almost complete overview of the foreign exposures of the 61 European banks. For only a small portion of foreign exposures – 3.6% of the total foreign exposures or 1.1% of the total assets – we do not know to which region or country these belong. This is the case when banks report their remaining foreign exposures as “other” without mentioning the countries belonging to this group. Table 1 also shows the percentage of total cross-border exposures per bank that could not be allocated to a specific country or region.

## Conflict of Interest

The authors declare that they have no known competing financial interests or personal relationships which have, or could be perceived to have, influenced the work reported in this article.

## Appendix Supplementary materials

Image, application 1
